# Healthcare Utilization and Costs Reduction after Radiofrequency Ablation For Atrial Fibrillation in the Brazilian Private Healthcare System

**DOI:** 10.5935/abc.20190139

**Published:** 2019-08

**Authors:** Eduardo Benchimol Saad, Daiane Oliveira Tayar, Rodrigo Antonini Ribeiro, Silvio Mauro Junqueira Jr., Priscila Andrade, Andre d'Avila

**Affiliations:** 1 Hospital Pró-Cardíaco - Eletrofisiologia, Rio de Janeiro, RJ - Brazil; 2 Johnson and Johnson Medical Brasil - Departamento de Economia da Saúde e Mercado de Acesso, São Paulo, SP - Brazil; 3 HTANALYZE - Economia da Saúde, Porto Alegre, RS - Brazil; 4 Hospital SOS Cardio - Serviço de Arritmia Cardíaca, Florianópolis, SC - Brazil

**Keywords:** Catheter Ablation, Arrhythmias Cardiacs, Hospitalization, Hospital Costs, Atrial Fibrillation, Care Costs/trends

## Abstract

**Background:**

Atrial fibrillation (AF) is the most common arrhythmia worldwide, with significantly associated hospitalizations. Considering its growing incidence, the AF related economic burden to healthcare systems is increasing. Healthcare expenditures might be substantially reduced after AF radiofrequency ablation (AFRA).

**Objective:**

To compare resource utilization and costs before and after AFRA in a cohort of patients from the Brazilian private healthcare system.

**Methods:**

We conducted a retrospective cohort study, based on patients’ billing information from an administrative database. Eighty-three adult patients who had an AFRA procedure between 2014 and 2015 were included. Healthcare resource utilization related to cardiovascular causes, including ambulatory and hospital care, as well as its costs, were analyzed. A p-value of less than 0.05 was considered statistically significant.

**Results:**

Mean follow-up was 14.7 ± 7.1 and 10.7 ± 5.4 months before and after AFRA, respectively. The 1-year AF recurrence-free rate was 83.6%. Before AFRA, median monthly total costs were Brazilian Reais (BRL) 286 (interquartile range [IQR]: 137-766), which decreased by 63.5% (p = 0.001) after the procedure, to BRL 104 (IQR: 57-232). Costs were reduced both in the emergency (by 58.6%, p < 0.001) and outpatient settings (by 56%, p < 0.001); there were no significant differences in the outpatient visits, inpatient elective admissions and elective admission costs before and after AFRA. The monthly median emergency department visits were reduced (p < 0.001).

**Conclusion:**

In this cohort, overall healthcare costs were reduced by 63.5%. A longer follow-up could be useful to evaluate if long-term cost reduction is maintained.

## Introduction

Atrial fibrillation (AF) is a public health problem. Estimates of incidence and prevalence vary worldwide.^1^ AF incidence will rise from 1.2 million cases per year in 2010 to 2.6 million cases in 2030; in the same period, prevalence will increase from 5.2 million to 12.1 million.^2^ In Brazil, estimates are less clear; a recent study showed a prevalence of 1.8% in the general population.^3^ However, considering the ageing of the population in rapidly developing countries such as Brazil, this number will probably increase in the near future.^4^

The disease is associated with high healthcare expenditures. In the USA, the annual cost of AF was an estimated US$26 billion, while in the Euro Heart Survey the estimated combined annual cost in 5 countries (Greece, Italy, the Netherlands, Poland and Spain) was €6.2 billion.^4^ Such expenditures represent a large economic burden: AF is estimated to contribute with more than 1% of total healthcare costs in projections made in 10 high-income countries.^5^ The clinical burden is also significant, especially relating to stroke: about a third of patients with the cerebrovascular disease have AF, which in turn incurs in a greater probability of a larger stroke area in brain imaging exams and, therefore, worse prognosis.^6-8^

Catheter ablation is an established treatment option for restoration of sinus rhythm, which can increase the quality of life and possibly lead to health care expenditure savings in the long term.^9,10^ The reduction in resource consumption and costs can be seen already in the first year of the procedure, and this is maintained in the following years.^11^ Even considering the cost of the procedure, it can lead to total healthcare costs reduction after 2 years, especially in younger patients.^12-15^

To date, there is scarce data of the economic impact of catheter ablation in middle-income countries, such as Brazil. The aim of this study was to compare medical costs and ambulatory and hospital service use before and after catheter ablation in a cohort of Brazilian AF patients treated in the private healthcare system.

## Methods

### Study design and dataset

This was a retrospective cohort study. The dataset used for the analyses was a patients’ reimbursement information from Orizon which contains a date-stamped log of all billed items by the cost-accounting department, including medications (only in-hospital use); laboratory, diagnostic, and therapeutic services; and primary and secondary diagnoses for each patient’s hospitalization. Both ambulatory and inpatient resource utilization are available in the dataset. About 12 million patients - which accounts from approximately 25% of patients in the Brazilian private healthcare system - are included in the Orizon patients’ billings databases. No informed consent was required because all data were from the patients' reimbursement information and their personal information was anonymous.

All adult patients (over 18 years old) who had a hospital admission between January 2014 and December 2015 and underwent catheter ablation with an ICD-10 code of AF (I48) were potentially eligible for the current analysis. The following eligibility criteria must have been met for patient inclusion in the current analysis:

- Elective radiofrequency ablation procedure, with a previous three-dimensional electrophysiologic mapping;
- Available age, gender and ICD code information;
- No registry of previous ablation procedures in the dataset;
- Use of point by point ablation (standard irrigated, irrigated with contact force sensors and non-irrigated);
- Minimum of 3 months of follow-up before and after the ablation procedure.

Outcomes were evaluated both in the perioperative admission as well as in any readmission that occurred up to 2 years after the ablation procedure.

### Study variables

The following variables were evaluated for each patient: age, gender, comorbidities (such as ischemic heart disease [IHD], chronic heart failure [CHF] and conduction disorders, among others), perioperative complications, short- and long-term AF recurrence-free rate, cardiovascular events, healthcare resources utilization (including ambulatory and emergency care) and costs. Details regarding variable definitions of these variables are described in the next paragraphs.

Comorbidities were defined according to ICD-10 codes registered in the ambulatory and emergency visits from the patients in the database. AF recurrence was defined when a new ablation or a cardioversion procedure was performed or upon resumption of antiarrhythmic drug use in the follow-up period, after the three-month blanking period. The cardiovascular events evaluated (both in the pre- and post-procedural follow-up) were: acute coronary syndromes (ACS), stroke and arrhythmias. ACS was defined whenever a patient had requests for electrocardiogram plus either troponin or MB fraction of creatine kinase (CK-MB), as well as one of the following, billed items: any thrombolytic, angioplasty procedure, or a combination of medications highly suggestive of ACS (such as any form of heparin, antiplatelet drugs, nitrates, and statins). Ischemic stroke was defined when a patient had a request of either a computerized tomography or nuclear magnetic resonance of the brain, a prescription of antiplatelet agent or low-molecular-weight heparin, and billing of exams such as an echocardiogram, carotid doppler ultrasound, and an intensive care unit (ICU) admission. Hemorrhagic stroke was defined when a patient had a brain imaging exam (magnetic resonance or computerized tomography) and a compatible ICD-10, and admission to ICU. Arrhythmic events were defined when there were billed items related either to: electric cardioversion, internal cardioverter-defibrillator implantation, ablation procedure, surgical correction of arrhythmia, or prescription of in-hospital antiarrhythmic drugs suggestive of an acute arrhythmic event in patients where and electrocardiogram was also requested.

The use of resources and their related costs were computed by summing all billed items (both ambulatory and emergency/in-hospital care). Only cardiovascular related resources and costs were computed. To calculate mean monthly costs, we divided total costs by the number of follow-up months. Costs were further divided into ambulatory care, emergency related and elective admissions.

### Statistical analysis

Continuous variables are presented as mean and standard deviation (SD) when they followed a normal distribution, and as median and interquartile range (IQR) when the distribution was non-normal. However, considering that cost (expressed as Brazilian Reais [BRL]) is usually a non-normal variable, but it is interesting to know the mean value since the total costs of any given sample of patients is equal to its mean times the total number of individuals, we present cost data in both ways. Categorical variables are presented as absolute values and proportions.

Comparison between variables employed the Wilcoxon test for non-normally distributed variables and the paired student T-test for the ones with normal distribution. Fisher’s exact test was used to compare categorical variables between groups. The AF recurrence-free rate was evaluated with the Kaplan Meier methods. In the evaluation of possible predictors of better event-free survival, we used the log-rank test. When the same predictors were analyzed regarding their impact on the before-and-after cost difference, the Mann-Whitney test was employed. All analyses were performed using SPSS version 20.0. A p-value of less than 0.05 was considered statistically significant.

## Results

**Figure 1:**
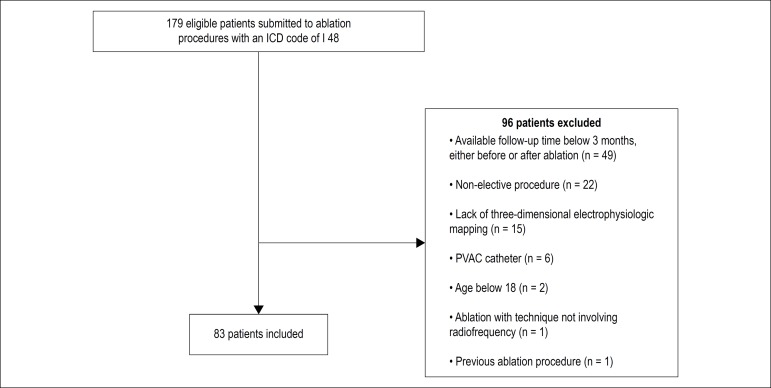
Flow chart of patient selection

Among 179 potentially eligible patients, 83 fulfilled the eligibility criteria and were included in the analysis (Figure 1).

Demographics and perioperative patients’ data are presented in Table 1. Approximately 70% of the study sample was comprised of male patients, with a mean age of 52.8 years (SD: 14.6). The most common comorbidities were hypertension (18%) and IHD (12%).

**Table 1:** Demographics and perioperative information of study patients

Variable	Total (%)
Male gender	58 (69.9)
Age*	52.8 (14.6)
**Comorbidities**	
Hypertension	15 (18)
Heart failure	5 (6)
Ischemic heart disease	10 (12)
Valvular heart disease	4 (4.8)
Conduction system disease	3 (3.6)
Diabetes	4 (4.8)
Sleep apnea	7 (8.49)
Thyreoid disease	5 (6)
Pre-procedural follow-up time (months)*	14.4 (7.2)
Post-procedural follow-up time (months)*	10.9 (5.4)
Prucedural LOS (days)*	1.93 (1.6)
Catheter cost	11,468 (4,591)

* Mean ± standard deviation.

In one year, the success rate was 83.6%. In the evaluation of possible predictors of longer event-free rate, none of the comorbidities investigated (hypertension, heart failure, and ischemic or valvular heart disease) was associated with this outcome (p > 0.05 for all variables in the log-rank test). Only one patient suffered peri-procedural complications (a hemorrhagic stroke).

Table 2 presents monthly resource use and costs before and after the ablation procedure. The monthly median number of emergency department visits reduced from 0.10 (IQR: 0.04 - 0.23) in the pre-ablation period to 0 (IQR: 0 - 0.11) in the post-ablation period (p < 0.001). Median monthly total costs had a 68.5% decrease, from 330.95 (IQR: 142.36 - 754.17) to 104.21 (IQR: 56. 35 - 226,51, p < 0.001). Outpatient and emergency-related costs were also reduced, by 48.8% and 100%, respectively (p < 0.001 for both variables). The monthly number of elective hospital admissions and its related costs, as well as outpatient office visits, did not have a statistically significant change between pre- and post-ablation periods.

**Table 2:** Monthly resource use and costs before and after the ablation procedure

Outcome	Before ablation - mean (SD)	Before ablation - median (IQR)	After ablation - mean (SD)	After ablation - median (IQR)	p value
Number of outpatient office visits	0.05 (0.15)	0 (0 - 0)	0.04 (0.10)	0 (0 - 0)	0.770
Number of emergency department visits	0.17 (0.21)	0.10 (0.04 - 0.23)	0.08 (0.16)	0 (0 - 0.11)	< 0.001
Number of emergency department visits - arrhythmic ICD	0.05 (0.07)	0 (0 - 0.09)	0.01 (0.04)	0 (0 - 0)	< 0.001
Number of elective hospital admissions	0.01 (0.02)	0 (0 - 0)	0.01 (0.04)	0 (0 - 0)	0.134
Total costs (BRL)	747.75 (1,315.38)	330.95 (142.36 - 754.17)	589.93 (1,779.83)	104.21 (56,35 - 226,51)	< 0.001
Outpatient costs (BRL)	156.81 (161.90)	121.48 (56.35 - 206.87)	83.74 (95.17)	62.70 (32.91 - 105.15)	< 0.001
Emergency related costs (BRL)	500.95 (1,268.61)	65.21 (3.54 - 433.88)	110.57 (358.86)	0 (0 - 36.98)	< 0.001
Elective admissions related costs (BRL)	89.99 (416.33)	0 (0 - 0)	395.61 (1,720.18)	0 (0 - 0)	0.215

SD: standard deviation; IQR: interquartile range; BRL: Brazilian Reais. P values were calculated with non-parametric tests since all variables had a non-normal distribution.

In the analysis of variables associated with a greater reduction in total monthly cost after the ablation procedure, none of the comorbidities evaluated - hypertension, heart failure, and ischemic or valvular heart disease - showed statistical significance (p > 0.10 for all variables).

## Discussion

In this study, we found that catheter ablation resulted in reduced ambulatory and hospital care costs during a mean post-procedural follow-up of 10.7 months, with a monthly median cost reduction of 68.5%: from BRL 330.95 before to BRL 104.21 after the procedure. Cost reduction occurred both in the outpatient setting (from BRL 121.48 to BRL 62.70) and in the emergency-related component (from BRL 65.21 to BRL 0). The procedure presented a success rate of 83.6% after 1 year of follow up which is compatible with recent studies conducted elsewhere using contact-force catheters.^13,14^ The number of serious complications was 1.2%, which is not different from other small cohorts in the literature.^16,17^

Other reports from the literature have also seen the impact of post-ablation cost reduction. In the larger study published to date, Ladapo et al.^11^ included 3,194 patients from administrative databases in the US.^11^ In that research, the approach was slightly different: they considered that costs can actually increase in the 6 months following the procedure, as a result of the need of reablation in a fraction of the sample, as well as the treatment of peri-procedural complications. Therefore, they analyzed the period from 6 to 36 months after ablation, divided into 6-month cycles. In the time frame of 6-12 months after ablation, mean monthly costs reduced around US$ 800, in comparison with the 6 months immediately before ablation. This number reduced until 18-24 months (where the reduction, compared to before ablation, was around US$ 200), and then increased again to around US$ 800 in the 30-36 months period. However, only 1/3 and 1/10 of patients had at least 24 months and 36 of follow-up time, respectively, making this long-term data more imprecise. Regardless, it seems considerably robust that cost reductions are noted already in the first year, and that it is retained over a longer follow-up period.

Some studies in the literature have estimated how long after catheter ablation the procedure would become “cost-neutral”. In a French retrospective cohort study that included 118 consecutive patients submitted to radiofrequency ablation for paroxysmal AF during a mean follow-up of 32 ± 15 weeks, it was estimated that from the 5^th^ year onwards, total accumulated costs would be smaller in patients submitted to ablation, as compared to medical treatment.^14^ In two Canadian economic models, the cost-neutrality would occur between 2 and 4 years of follow-up.^13,15^ These three studies, however, were not fully based on collected data and included some future projections and modelling.

Some limitations of our study must be acknowledged. The dataset used for all analyses was based on patient billing information and the patients were made anonymous to the researches. Therefore, direct contact to establish the recurrence was not possible. This could overestimate the success rate because the recurrence was only based on the use of healthcare resources (use of antiarrhytmic drug in the emergency room, cardioversion or repeated procedures) or indirectly by the purchase of antiarrhythmic drug in the pharmacies by the patient. The use of an administrative database carries the risk of bias as any retrospective study, as well as the problems associated with the lack of individual clinical patient information. Moreover, we did not included costs with ambulatory medications, since this information was not available in the patients’ billings information dataset, which did not included out-of-pocket patients expenditures. Finally, the sample size was not large, and the analysis of possible predictors of greater cost reductions after the ablation procedure was probably underpowered.

## Conclusion

In this sample of patients from the Brazilian private healthcare sector, catheter ablation of AF was associated with significantly decreased costs - both ambulatory and hospital-based.
